# Acute Cordyceps militaris supplementation and elevated resting oxygen uptake with faster reaction times: A randomized crossover trial

**DOI:** 10.1371/journal.pone.0351725

**Published:** 2026-07-15

**Authors:** Hamidreza Farzan, Maryam Koushkie Jahromi

**Affiliations:** Department of Sports Sciences, School of Education and Psychology, Shiraz University, Shiraz, Iran; ICAR-Directorate of Mushroom Research, INDIA

## Abstract

**Background:**

Cordyceps militaris (CM) demonstrates neuroprotective properties in chronic preclinical models, but its acute effects on cognitive and physiological parameters during exhaustive exercise remain uncharacterized in humans.

**Study design:**

In a randomized, double-blind, placebo-controlled crossover trial with a 7-day washout, 12 recreationally active young men (mean age 21.6 ± 2.9 years, VO₂max: 38.6 ± 5.5 mL·kg ⁻ ¹·min ⁻ ¹) ingested 1 g of standardized CM extract or a cornstarch placebo 30 minutes before a maximal cycling test to exhaustion. Cognitive performance (Stroop reaction time and accuracy) and physiological measures (oxygen uptake, blood glucose, blood pressure, and heart rate) were assessed pre-exercise and 3 minutes’ post-exercise.

**Results:**

Stroop reaction time exhibited a significant main effect of Condition (F(1,11) = 5.45, p = 0.040) and Time (F(1,11) = 16.37, p = 0.002), with faster responses overall in the CM condition. Both conditions improved from pre- to post-exercise (placebo: −148.92 ms; CM: −104.67 ms). The Condition × Time interaction was non-significant (F(1,11) = 1.01, p = 0.337). No between-group difference was observed at pre-exercise (p = 0.167), but CM produced faster reaction times at post-exercise (963.92 ± 84.99 ms vs. 1034.33 ± 84.16 ms; p = 0.042, d = 0.88). Accuracy remained near-ceiling (>99%) with no condition differences. CM elevated resting VO₂ (0.37 vs. 0.24 L/min; p = 0.022) without altering peak exercise VO₂ (p = 0.490). No significant Condition effects or interactions were observed for blood glucose, blood pressure, or heart rate (all p > 0.05).

**Conclusion:**

Acute CM supplementation improved post-exercise reaction time and elevated resting oxygen uptake, but did not alter the magnitude of exercise-induced changes in cognitive or physiological parameters. This suggests CM exerts an acute cognitive benefit independent of exercise modulation.

## Introduction

Exhaustive exercise imposes acute metabolic stress that transiently compromises cognitive processing speed—a critical limitation in athletic and tactical settings where rapid decision-making under fatigue determines performance outcomes [1,2]. While moderate exercise typically enhances cognition via increased cerebral perfusion and neurotrophic signaling, maximal exertion triggers dynamic physiological shifts—including transient alterations in glucose availability, autonomic activation, and substrate utilization—that can differentially affect cognitive domains [3]. Notably, cognitive responses to exhaustive exercise are context-dependent: some protocols reveal transient improvement [4], whereas others demonstrate practice-related improvements or arousal-induced enhancements when assessments occur shortly post-exercise [5,6]. This variability underscores the importance of characterizing cognitive-physiological relationships across specific timepoints rather than assuming uniform impairment.

Cordyceps militaris (CM) a medicinal fungus rich in cordycepin (3′-deoxyadenosine) and β-glucans, has demonstrated ergogenic and neuroprotective properties in preclinical models [7]. Mechanistically, cordycepin functions as an adenosine analog that is phosphorylated intracellularly to cordycepin monophosphate, an AMP-mimetic that activates AMP-activated protein kinase (AMPK) [8]. AMPK activation enhances mitochondrial oxidative capacity and substrate flexibility—effects that could stabilize cerebral energy supply during metabolic stress [9]. Preclinical evidence for CM’s cognitive benefits presents a critical temporal dichotomy. The majority of animal studies demonstrating neuroprotection employ chronic administration paradigms (days to weeks), inducing structural adaptations including hippocampal neurogenesis, reduced neuronal apoptosis, and mitochondrial biogenesis via sustained AMPK/PGC-1α signaling [1,10,11]. These chronic mechanisms operate on timescales fundamentally incompatible with acute supplementation—structural remodeling cannot occur within 30 minutes of a single dose. Acute cognitive effects—if they exist—must operate via distinct rapid-signaling pathways. AMPK activation occurs within minutes of cordycepin exposure in skeletal muscle [8], Only one preclinical study to date has demonstrated acute cognitive preservation during exercise stress: Cheng et al. (2025) reported that a single cordycepin dose administered immediately before exhaustive running preserved learning/memory in mice [1]—a finding suggesting rapid metabolic stabilization may buffer neural function during acute stress. However, human cordycepin pharmacokinetics remain poorly characterized; while rodent data suggest peak plasma concentrations at 25–35 min post-ingestion [1], bioavailability following oral CM extract in humans is unverified.

Human trials of CM have predominantly examined chronic supplementation effects on aerobic capacity or general wellness [12] leaving acute cognitive-metabolic effects in exercise contexts poorly characterized. Existing meta-analyses addressing CM’s cognitive effects focus exclusively on chronic administration in aging or disease models [13,14], contexts fundamentally different from acute metabolic challenges in healthy young adults.

Acute maximal exercise elicits profound physiological changes that are critical for energy mobilization and adaptation to stress. These responses include dynamic alterations in blood glucose levels, blood pressure, oxygen uptake (VO₂), and heart rate [13] which may be mediated by exercise impact on autonomic function [14]. Optimal glucose availability is crucial for brain function [15], and cardiovascular responses directly affect cerebral blood flow [16] both of which may influence cognitive processes depending on timing and magnitude of perturbation. Moreover, no human study has integrated metabolic mediators (resting VO₂ as a proxy for basal oxidative capacity; glucose kinetics as an index of substrate stability) with cognitive outcomes during the immediate post-exercise recovery window when cerebral energy deficits peak.

Therefore, this study aimed to examine whether acute CM supplementation influences cognitive performance and key physiological parameters—including blood glucose, blood pressure, oxygen uptake (VO₂), and heart rate—at rest and following exhaustive exercise in recreationally active young men. Rather than presupposing a specific directional effect on exercise-induced cognitive change, we sought to characterize CM’s acute profile across the physiologic–cognitive interface during metabolic stress. Findings may inform understanding of acute nootropic effects of medicinal fungi and their potential applications in contexts requiring rapid cognitive processing.

## Materials and methods

All procedures were conducted in accordance with the Declaration of Helsinki and approved by the Ethics Committee of Shiraz University, Faculty of Physical Education and Sport Sciences (approval code: IR.US.PSYEDU.REC.1404.069).

### Participants

Twelve physically active young male volunteers were recruited from the local university community. Participants’ mean estimated peak oxygen uptake (VO₂max), calculated from the maximal cycling test, was 38.6 ± 5.5 mL·kg ⁻ ¹·min ⁻ ¹, consistent with a recreationally active fitness level. Elite athletes exhibit high metabolic adaptations (e.g., enhanced glycogen sparing, cerebral perfusion stability) [17,18] that could mask acute supplement effects. Recreational exercisers experience pronounced transient metabolic perturbations during exhaustive exercise—making them an ideal population to test whether CM stabilizes the cerebral energy supply-demand balance under stress. Inclusion criteria were: (1) age 18–30 years; (2) self-reported recreational physical activity ≥150 min/week; (3) no smoking or excessive caffeine use (>400 mg/day); (4) no history of cardiovascular, metabolic, or neurological disease; and (5) no use of ergogenic, cognitive-enhancing, or anti-inflammatory supplements for ≥4 weeks prior to testing. Exclusion criteria included BMI < 18.5 or >30 kg/m² and unstable body weight (±3 kg) in the preceding month.2.2. Participants’ mean estimated peak oxygen uptake (VO₂max), calculated from the maximal cycling test, was 38.6 ± 5.5 mL·kg ⁻ ¹·min ⁻ ¹, consistent with a recreationally active fitness level.

An a priori power analysis was conducted using G*Power (version 3.1.9.7). Based on a prior acute nootropic supplementation study reporting a medium-to-large effect on executive function (Cohen’s f = 0.35) [19], a repeated-measures ANOVA (within factors, two groups, two measurements) with α = 0.05 and power (1 – β) = 0.80 indicated a required sample size of 10 participants. To account for potential attrition and variability in responses, we recruited 12 participants. While adequately powered to detect main effects, the study may be underpowered to detect smaller interaction effects, which should be considered when interpreting non-significant Condition × Time outcomes.

Participant recruitment and research conduction for this study took place from 10.07.2025 to 20.09. 2025. All participants provided written informed consent prior to any study procedures. The consent form detailed the study’s purpose, procedures, potential risks and benefits, and the right to withdraw at any time without penalty.

### Study design

This study employed a randomized, double-blind, placebo-controlled, crossover design. Each participant completed two experimental visits separated by a 7-day washout period to eliminate potential carryover effects. Randomization was performed by an independent researcher using computer-generated block randomization (block size = 4). Participants were allocated to one of two supplementation sequences: CM→Placebo or Placebo→CM (n = 6 per sequence).

Blinding was maintained at all levels:

*Supplement Preparation:* Capsules were prepared and coded (A/B) by a third-party technician not involved in recruitment or testing.

*Participant & Investigator Blinding:* Participants, test administrators, and outcome assessors were unaware of capsule identity throughout data collection.

*Data Analysis Blinding:* The data analyst remained blinded to condition codes until completion of the statistical analysis.

*Blinding Procedure:* The blinding key was held by the independent researcher and was only revealed after database lock.

Participants were instructed to maintain their habitual diet and physical activity patterns and to abstain from alcohol, caffeine, and strenuous exercise for 24 h before each testing session.

### Supplementation protocol

In the Cordyceps militaris (CM) condition, participants ingested a single 1 g dose of CM fruiting body extract (Real Mushrooms®, product code RM-CM150C, Lot # C24156001-1), standardized to contain ≥150 mg/g (15% w/w) β-glucans and ≥2.5 mg/g (0.25% w/w) cordycepin, as verified by third-party HPLC (Certificate of Analysis available at: https://realmushrooms.com/coa/cm150c; see Supplementary S1 File for full analytical report). In the placebo (PLA) condition, participants received an identical-appearing 1 g capsule filled with pharmaceutical-grade cornstarch, confirmed via HPLC to be free of detectable cordycepin, adenosine, or β-glucans. Capsules were administered orally with 200 mL water 30 min prior to baseline testing — a timing selected to align with estimated peak plasma cordycepin concentrations [1]. Compliance was verified via verbal confirmation and capsule count.

The 1 g dose was selected based on: (1) standardization to deliver ≥150 mg β-glucans and ≥2.5 mg cordycepin per serving; (2) alignment with human supplementation literature reporting biological activity of CM extracts within the 1–4 g/day range for acute protocols [20–22]; (3) preclinical pharmacokinetic studies in rats indicating cordycepin undergoes rapid distribution and elimination, with plasma levels declining substantially within ~30 minutes of administration [23]; and (4) practical considerations for tolerability and feasibility within a randomized crossover design.

### Experimental procedures

All trials occurred between 08:00 and 12:00 h to control for circadian influences. Upon arrival, participants had consumed a standardized breakfast 2 h earlier (~650 kcal: 250 g boiled potatoes [≈59 g CHO, 5 g PRO, 0.2 g FAT] and 3 large boiled eggs [0 g CHO, 18 g PRO, 15 g FAT]; values from USDA FoodData Central). After 10 min seated rest, baseline assessments were conducted in fixed order: (1) capillary blood glucose (Accu-Chek Active), (2) seated systolic/diastolic blood pressure (manual sphygmomanometer), (3) resting heart rate (radial pulse ×15 s × 4), (4) resting oxygen uptake (VO₂rest; Cortex Metalyzer 3B), and (5) Stroop task (25-item incongruent trials, Farsi version). Participants then performed an incremental cycling test to volitional exhaustion on a Monark 839E cycle ergometer: starting at 50 W, workload increased by 25 W every 2 min at 50 rpm; exhaustion defined as inability to maintain ≥45 rpm for >5 s despite standardized verbal encouragement. At precisely 3.0 ± 0.1 min post-exhaustion (timed by digital stopwatch), the identical post-exercise assessment battery was repeated.

All physiological variables (VO₂, glucose, BP, HR) were selected a priori as mechanistic mediators hypothesized to link CM’s metabolic effects to cognitive outcomes. Specifically: Resting VO₂: Proxy for basal mitochondrial oxidative capacity (AMPK-mediated); Blood glucose kinetics: Index of substrate stability for neural tissue; BP/HR: Controls to confirm effects are metabolic (not hemodynamic) in origin.

### Instruments

All devices were calibrated per manufacturer protocols before each session. VO₂ was measured via breath-by-breath indirect calorimetry (Cortex Metalyzer 3B, Leipzig, Germany; calibrated with certified gases). Blood glucose was assessed from fingertip capillary samples using a Roche Accu-Chek Active glucometer (precision: ± 15% per ISO 15197:2013). Blood pressure was measured manually using a calibrated mercury-free sphygmomanometer (Glamour, China) with appropriate cuff size. Heart rate was determined manually via radial pulse palpation for 15 s and multiplied by 4 (validated against Polar H10 ECG in pilot testing).

The Stroop Color–Word Interference Test was administered via a custom Android application developed in Farsi, modeled on the standardized incongruent trial format (Golden, 1978). The task consisted of 25 incongruent trials. This abbreviated format was selected to minimize participant fatigue and testing time during the acute post-exercise window, and has shown strong internal consistency in pilot validation (Cronbach’s α = 0.91 for reaction time, 0.88 for accuracy) in a separate Farsi-speaking sample (n = 10). To minimize practice effects, all participants completed a familiarization session with 15 practice trials one week prior to the first experimental visit.

### Statistical analysis

Analyses were performed using SPSS v26.0 (IBM Corp., USA). Normality was assessed via Shapiro–Wilk tests and all variables met the assumption of normality (p > 0.05). A two-way repeated-measures analysis of variance (ANOVA) with factors for Condition (CM vs. Placebo) and Time (Pre- vs. Post-exercise) was used to examine main effects and interactions for all outcomes. Where significant main or interaction effects were detected, Bonferroni-adjusted pairwise comparisons were conducted to examine specific differences between conditions and time points. Effect sizes were reported as partial eta-squared (η²ₚ) for ANOVA effects and as Cohen’s d with 95% confidence intervals for pairwise comparisons. Statistical significance was defined as p < 0.05 for the primary outcome.

## Results

### Participant flow

**Fig 1:**
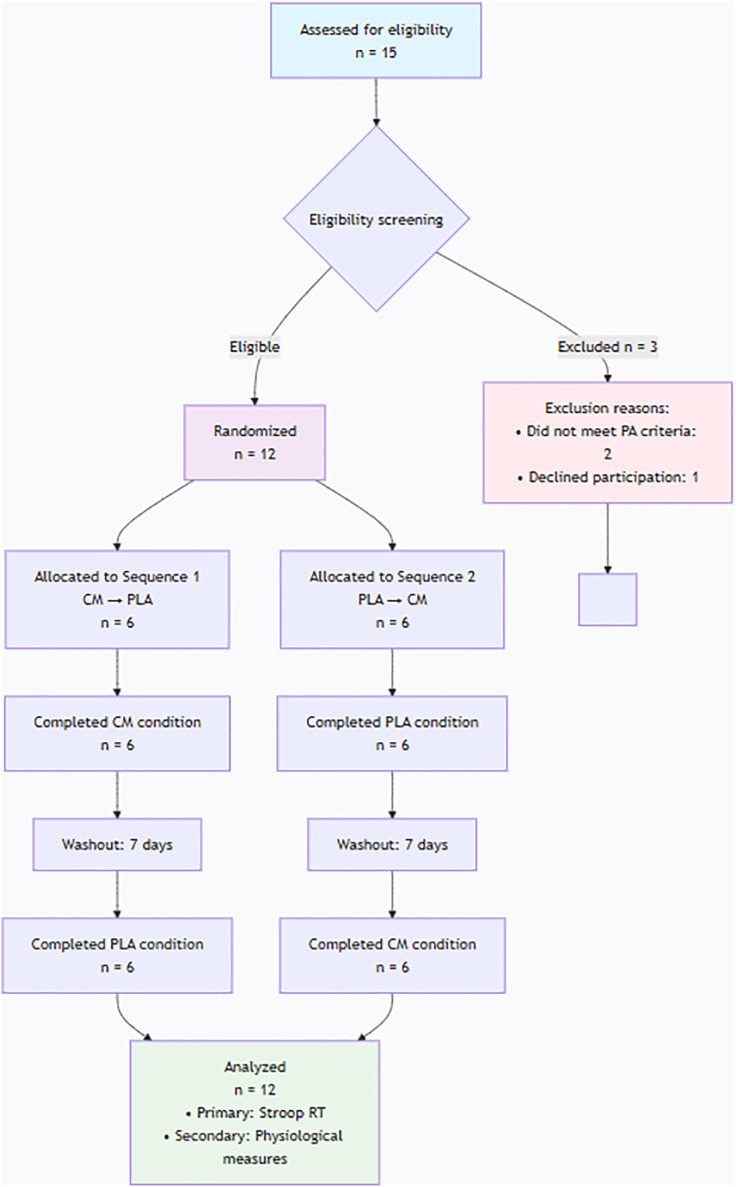
CONSORT flow diagram of participant recruitment, randomization, allocation, and analysis through the randomized, double-blind, placebo-controlled crossover trial.

Fifteen individuals were assessed for eligibility; three were excluded (two did not meet physical activity criteria; one declined participation after consent). Twelve recreationally active young men (mean age: 21.6 ± 2.9 years; height: 179.9 ± 5.7 cm; weight: 84.2 ± 14.8 kg; BMI: 26.0 ± 4.1 kg/m²) were randomized and completed both experimental conditions with no dropouts, protocol deviations, or adverse events. Estimated peak oxygen uptake was 38.6 ± 5.5 mL·kg ⁻ ¹·min ⁻ ¹. All participants were included in the intention-to-treat analysis (Fig 1).

### Cognitive function (Stroop Test)

#### Stroop reaction time.

A two-way repeated-measures ANOVA revealed a significant main effect of Condition (F(1,11) = 5.45, p = 0.040, η²ₚ = 0.331) and a significant main effect of Time (F(1,11) = 16.37, p = 0.002, η²ₚ = 0.598). The Condition × Time interaction was not significant (F(1,11) = 1.01, p = 0.337, η²ₚ = 0.084), indicating that CM did not differentially modulate the magnitude of exercise-induced change in reaction time.

**Fig 2:**
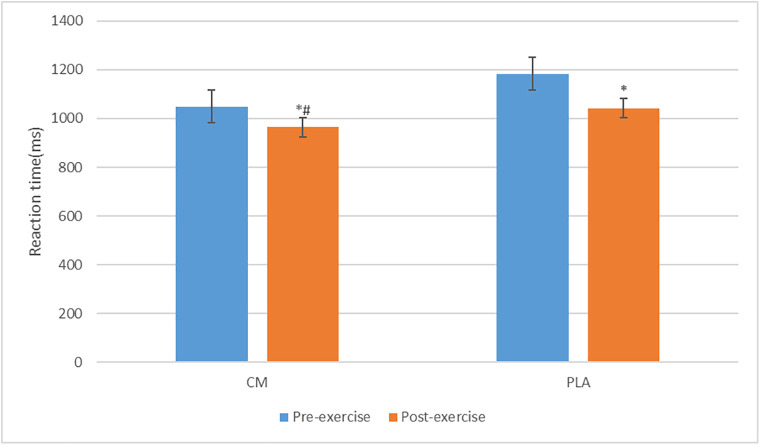
Stroop task reaction time by condition and time point. Mean reaction times (milliseconds) are displayed for Placebo (PLA) and Cordyceps militaris (CM) conditions at Pre-Exercise and Post-Exercise. Error bars represent standard deviations. Significant decrease from Pre- to Post-Exercise in both conditions (p < 0.01). #Significantly faster reaction time with CM compared to PLA at Post-Exercise (p = 0.042).

Post-hoc comparisons confirmed a significant decrease from pre- to post-exercise in both conditions (both p < 0.01). While no between-condition difference was present at pre-exercise (mean difference = −134.58 ms, 95% CI [−279.44, 10.28]; p = 0.167, d = 0.59), reaction times were significantly faster in the CM condition compared to placebo at post-exercise (mean difference = −78.75 ms, 95% CI [−140.54, −16.96]; p = 0.042, d = 0.83) (Table 1, Fig 2).

**Table 1:** Cognitive performance outcomes (Stroop test) at pre- and post-exercise for Placebo and Cordyceps militaris (CM) conditions.

Outcome	Time Point	Placebo (n = 12) Mean ± SD	CM (n = 12) Mean ± SD	Main Effects (ANOVA)	Pairwise Comparison (CM vs. Placebo)
**Reaction Time (ms)**	Pre-Exercise	1183.33 ± 201.37	1048.75 ± 166.11	**Condition:** F(1,11) = 5.45, p = 0.040, η²ₚ = 0.331 **Time:** F(1,11) = 16.37, p = 0.002, η²ₚ = 0.598 **Intx:** F(1,11) = 1.01, p = 0.337	Pre: p = 0.167, d = 0.59 **Post: p = 0.042, d = 0.83**
	Post-Exercise	1042.83 ± 79.52	**964.08 ± 78.63**		
**Accuracy (%)**	Pre-Exercise	98.40 ± 4.58	99.93 ± 0.16	**Condition:** F(1,11) = 0.17, p = 0.692 **Time:** F(1,11) = 0.33, p = 0.580 **Intx:** F(1,11) = 3.12, p = 0.105	Pre: p = 0.532 Post: p = 0.812
	Post-Exercise	99.60 ± 1.14	99.40 ± 1.24		

Data are mean ± standard deviation. SD = standard deviation; Intx = Condition × Time interaction. Significant p-values (< 0.05) are shown in bold. Effect sizes (Cohen’s d) for pairwise comparisons are provided where significant.

#### Stroop accuracy.

A two-way repeated-measures ANOVA revealed no significant main effect of Condition (F(1,11) = 0.17, p = 0.692, η²ₚ = 0.015), no significant main effect of Time (F(1,11) = 0.33, p = 0.580, η²ₚ = 0.029), and no significant Condition × Time interaction (F(1,11) = 3.12, p = 0.105, η²ₚ = 0.221).

**Fig 3:**
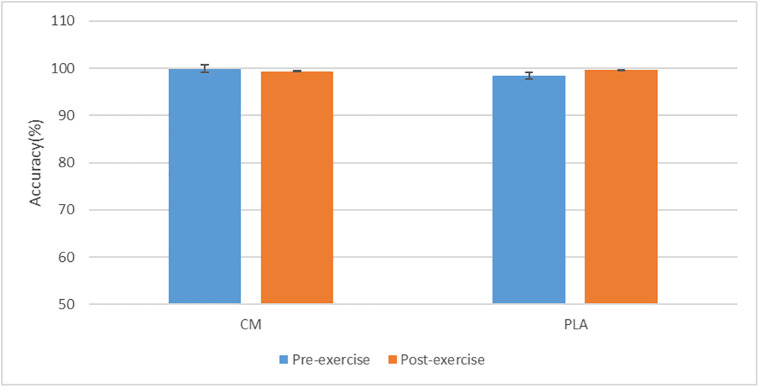
Stroop task accuracy by condition and time point. Accuracy (%) is shown for Placebo (PLA) and Cordyceps militaris (CM) conditions before (Pre-Exercise) and after (Post-Exercise) exhaustive exercise. Error bars represent standard deviations. No significant differences between conditions or time points were observed (p > 0.05).

Mean accuracy remained near-ceiling across all conditions and time points (range: 98.40% to 99.93%). Pairwise comparisons confirmed no significant between-condition differences at pre-exercise (mean difference = +1.53 percentage points, 95% CI [−0.64, 3.70]; p = 0.532) or post-exercise (mean difference = −0.20 percentage points, 95% CI [−1.64, 1.24]; p = 0.812). These near-ceiling values indicate that the observed reaction time improvements occurred without a speed-accuracy trade-off (Table 1, Fig 3).

### Physiological Measures

#### Oxygen Uptake (VO₂).

A two-way repeated-measures ANOVA revealed significant main effects of Condition (F(1,11) = 8.90, p = 0.013, η²ₚ = 0.447) and Time (F(1,11) = 705.02, p < 0.001, η²ₚ = 0.985), with no significant Condition × Time interaction (F(1,11) = 0.25, p = 0.627, η²ₚ = 0.022). As expected, VO₂ increased substantially from rest to peak exercise in both conditions (both p < 0.001). Resting VO₂ was significantly higher in the CM condition (4.06 ± 1.68 mL·kg ⁻ ¹·min ⁻ ¹) compared to placebo (2.80 ± 0.93 mL·kg ⁻ ¹·min ⁻ ¹); mean difference = +1.26 mL·kg ⁻ ¹·min ⁻ ¹ (95% CI [0.27, 2.25]), p = 0.022, d = 0.92.

**Fig 4:**
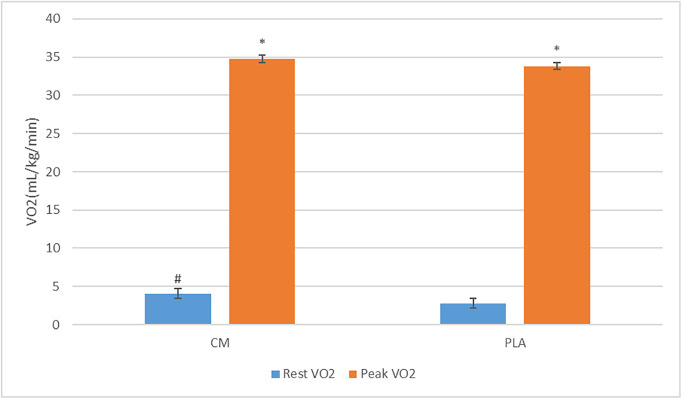
Resting and peak oxygen consumption (VO₂) by supplement condition and time point. Resting VO₂ was significantly higher following Cordyceps militaris (CM) versus placebo (PLA) (p = 0.022). Significant increase from Rest to Peak in both conditions (p < 0.001). *significant difference compared to pre exercise. # significant difference compared to placebo.

No significant between-condition difference was observed at peak exercise (CM: 34.75 ± 4.80 mL·kg ⁻ ¹·min ⁻ ¹ vs. placebo: 33.82 ± 4.15 mL·kg ⁻ ¹·min ⁻ ¹); mean difference = +0.93 mL·kg ⁻ ¹·min ⁻ ¹ (95% CI [−1.45, 3.31]), p = 0.490. (Table 2, Fig 4).

**Table 2:** Physiological outcomes at rest/pre-exercise and peak/post-exercise for Placebo and Cordyceps militaris (CM) conditions.

Outcome	Time Point	Placebo (n = 12) Mean ± SD	CM (n = 12) Mean ± SD	Main Effects (ANOVA)	Pairwise Comparison (CM vs. Placebo)
**VO₂ Rest (mL·kg ⁻ ¹·min ⁻ ¹)**	Rest	2.80 ± 0.93	**4.06 ± 1.68**	**Condition:** F(1,11) = 8.90, p = 0.013, η²ₚ = 0.447 **Time:** F(1,11) = 705.02, p < 0.001 **Intx:** F(1,11) = 0.25, p = 0.627	**Rest: p = 0.022, d = 0.92**
**VO₂ Peak (mL·kg ⁻ ¹·min ⁻ ¹)**	Peak Exercise	33.82 ± 4.15	34.75 ± 4.80		Peak: p = 0.490
**Blood Glucose (mg/dL)**	Pre-Exercise	100.30 ± 3.57	101.40 ± 3.28	**Condition:** F(1,11) = 0.37, p = 0.556 **Time:** F(1,11) = 66.31, p < 0.001 **Intx:** F(1,11) = 0.05, p = 0.835	Pre: p = 0.371 Post: p = 0.678
	Post-Exercise	93.93 ± 6.10	94.88 ± 4.20		
**Systolic BP (mmHg)**	Pre-Exercise	112.90 ± 7.32	115.58 ± 7.67	**Condition:** F(1,11) = 0.03, p = 0.876 **Time:** F(1,11) = 29.90, p < 0.001 **Intx:** F(1,11) = 0.29, p = 0.603	Pre: p = 0.963 Post: p = 0.821
	Post-Exercise	125.75 ± 9.29	127.10 ± 10.91		
**Diastolic BP (mmHg)**	Pre-Exercise	63.60 ± 5.74	60.50 ± 5.68	**Condition:** F(1,11) = 0.40, p = 0.540 **Time:** F(1,11) = 0.50, p = 0.494 **Intx:** F(1,11) = 4.14, p = 0.067	Pre: p = 0.211 Post: p = 0.758
	Post-Exercise	63.67 ± 4.05	64.50 ± 10.17		
**Heart Rate (bpm)**	Pre-Exercise	81.60 ± 10.74	81.83 ± 10.56	**Condition:** F(1,11) = 1.20, p = 0.296 **Time:** F(1,11) = 818.87, p < 0.001 **Intx:** F(1,11) = 0.00, p = 0.998	Pre: p = 0.422 Post: p = 0.428
	Post-Exercise	152.93 ± 10.58	157.88 ± 10.47		

Data are mean ± standard deviation. VO₂ = oxygen uptake; BP = blood pressure; bpm = beats per minute; SD = standard deviation; Intx = Condition × Time interaction. Significant p-values (< 0.05) are shown in bold. Effect sizes (Cohen’s d) are provided where pairwise comparisons were significant.

#### Blood glucose.

A two-way repeated-measures ANOVA revealed a significant main effect of Time (F(1,11) = 66.31, p < 0.001, η²ₚ = 0.858), with glucose declining from pre- to post-exercise in both conditions, reflecting the metabolic demands of exhaustive exercise. However, there was no significant main effect of Condition (F(1,11) = 0.37, p = 0.556, η²ₚ = 0.032) and no significant Condition × Time interaction (F(1,11) = 0.05, p = 0.835, η²ₚ = 0.004).

Pre-exercise blood glucose was comparable between CM (101.40 ± 3.28 mg/dL) and placebo (100.30 ± 3.57 mg/dL); mean difference = +1.10 mg/dL (95% CI [−1.35, 3.55]), p = 0.371.

Post-exercise blood glucose was also comparable between CM (94.88 ± 4.20 mg/dL) and placebo (93.93 ± 6.10 mg/dL); mean difference = +0.95 mg/dL (95% CI [−3.80, 5.70]), p = 0.678.

**Fig 5:**
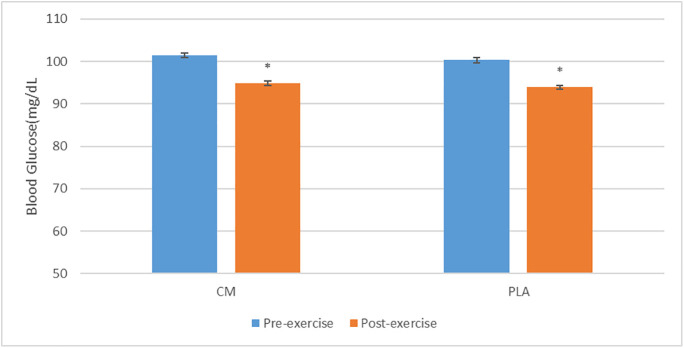
Blood glucose levels by supplement condition and time point. Measurements were taken at Pre-Exercise and Post-Exercise following ingestion of placebo (PLA) or Cordyceps militaris (CM). Error bars represent standard deviations. *Significant decrease from Pre- to Post-Exercise in both conditions (p < 0.001). No significant between-condition differences were observed.

These findings indicate that acute CM supplementation did not differentially influence glycemic responses compared to placebo (Table 2, Fig 5).

#### Blood Pressure.

Regarding systolic blood pressure, a two-way repeated-measures ANOVA revealed a significant main effect of Time (F(1,11) = 29.90, p < 0.001, η²ₚ = 0.731), with pressure increasing from pre- to post-exercise in both conditions, reflecting normal hemodynamic responses to maximal exertion. There was no significant main effect of Condition (F(1,11) = 0.03, p = 0.876, η²ₚ = 0.002) and no significant Condition × Time interaction (F(1,11) = 0.29, p = 0.603, η²ₚ = 0.025).

**Fig 6:**
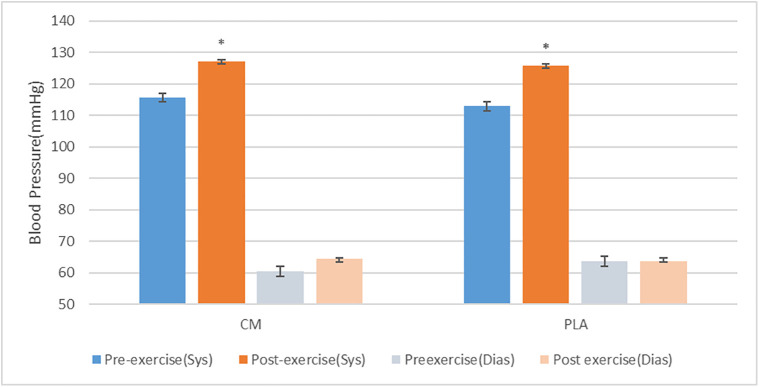
Systolic(Sys) and diastolic(Dias) blood pressure by supplement condition and time point. Measurements were taken at Pre-Exercise and Post-Exercise following ingestion of placebo (PLA) or Cordyceps militaris (CM). Error bars represent standard deviations. Systolic blood pressure increased post-exercise compared to pre-exercise. *Significant increase from Pre- to Post-Exercise in both conditions (p < 0.001). No significant between-condition differences were observed.

Pre-exercise systolic blood pressure was comparable between CM (115.58 ± 7.67 mmHg) and placebo (112.90 ± 7.32 mmHg); mean difference = +2.68 mmHg (95% CI [−6.38, 11.74]), p = 0.963. Post-exercise systolic blood pressure was also comparable between CM (127.10 ± 10.91 mmHg) and placebo (125.75 ± 9.29 mmHg); mean difference = +1.35 mmHg (95% CI [−8.28, 10.98]), p = 0.821. These findings indicate that acute CM supplementation did not differentially influence systolic blood pressure responses at rest or following exhaustive exercise (p = 0.821) (Table 2, Fig 6).

For diastolic blood pressure, a two-way repeated-measures ANOVA revealed no significant main effect of Condition (F(1,11) = 0.40, p = 0.540, η²ₚ = 0.035), no significant main effect of Time (F(1,11) = 0.50, p = 0.494, η²ₚ = 0.044), and no significant Condition × Time interaction (F(1,11) = 4.14, p = 0.067, η²ₚ = 0.273). Pre-exercise diastolic blood pressure was comparable between CM (60.50 ± 5.68 mmHg) and placebo (63.60 ± 5.74 mmHg); mean difference = −3.10 mmHg (95% CI [−8.13, 1.93]), p = 0.211. Post-exercise diastolic blood pressure was also comparable between CM (64.50 ± 10.17 mmHg) and placebo (63.67 ± 4.05 mmHg); mean difference = +0.83 mmHg (95% CI [−9.02, 10.68]), p = 0.758. These findings indicate that acute CM supplementation did not differentially influence diastolic blood pressure responses at rest or following exhaustive exercise (Table 2, Fig 6).

#### Heart rate.

A two-way repeated-measures ANOVA revealed a significant main effect of Time (F(1,11) = 818.87, p < 0.001, η²ₚ = 0.987), with heart rate increasing substantially from pre- to post-exercise in both conditions (placebo: + 71.33 bpm; CM: + 76.05 bpm). There was no significant main effect of Condition (F(1,11) = 1.20, p = 0.296, η²ₚ = 0.099) and no Condition × Time interaction (F(1,11) = 0.00, p = 0.998, η²ₚ = 0.000).

Pre-exercise heart rate was comparable between CM (81.83 ± 10.56 bpm) and placebo (81.60 ± 10.74 bpm); mean difference = +0.23 bpm (95% CI [−6.75, 7.21]), p = 0.422.

Post-exercise heart rate was also comparable between CM (157.88 ± 10.47 bpm) and placebo (152.93 ± 10.58 bpm); mean difference = +4.95 bpm (95% CI [−6.80, 16.70]), p = 0.428.

**Fig 7:**
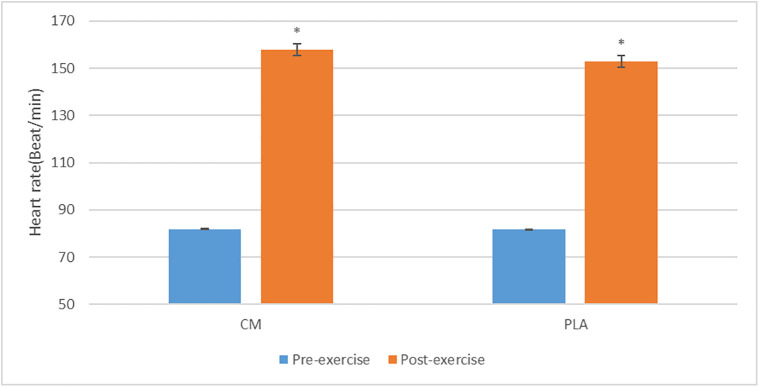
Heart rate by supplement condition and time point. Measurements were taken at Pre-Exercise and Post-Exercise following ingestion of placebo (PLA) or Cordyceps militaris (CM). Error bars represent standard deviations. *Significant increase from Pre- to Post-Exercise in both conditions (p < 0.001). No significant between-condition differences were observed.

These findings indicate that acute CM supplementation did not alter heart rate responses at rest or following exhaustive exercise (Table 2, Fig 7).

## Discussion

The present study examined the acute effects of CM supplementation on cognitive and physiological parameters at rest and following exhaustive exercise. The principal findings are threefold. First, acute CM administration produced faster Stroop reaction times compared to placebo, with the advantage persisting through the post-exercise assessment. Second, CM significantly elevated resting oxygen uptake without altering peak exercise VO₂, blood glucose, blood pressure, or heart rate. Third, the absence of significant Condition × Time interactions for all outcomes indicates that CM’s effect represents an elevation of baseline processing speed rather than modulation of exercise-induced changes. Collectively, these findings suggest that acute CM supplementation exerts rapid cognitive benefits through metabolic priming independent of hemodynamic or glycemic alterations.

### Effects of cordyceps on cognitive function

Acute CM administration produced a significant main effect on Stroop reaction time, with consistently faster responses across both timepoints compared to placebo. This effect manifested as a 70.42 ms advantage at post-exercise assessment, representing a 6.8% performance improvement. Accuracy remained near-ceiling (>99%) with no condition differences, indicating enhanced processing speed without speed-accuracy trade-offs.

Exhaustive exercise induced improvements in reaction time in both conditions, likely reflecting practice effects and exercise-induced arousal [24]. Critically, the Condition × Time interaction was non-significant, indicating CM did not differentially modulate the magnitude of pre-to-post exercise change. Rather than attenuation of impairment, CM’s effect represents an elevation of baseline processing speed that persisted through the post-exercise assessment—a finding relevant for contexts requiring sustained rapid decision-making irrespective of acute physical stressors.

Preclinical evidence provides context for CM’s effects on neural processing. In scopolamine-induced amnesia models, CM polypeptide significantly improved learning and memory performance, likely through antioxidant neuroprotection [25]. Similarly, cordycepin (3’-deoxyadenosine) administered for 3 weeks improved short-term spatial memory in Y-maze tests, accompanied by reduced hippocampal adenosine A₂A receptor density [26]. Methanol extracts of CM reversed scopolamine-induced memory deficits in rats while upregulating central cholinergic activity [7], and CM Condition improved memory after global cerebral ischemia while reducing hippocampal neuronal death [27]. These converging animal data establish Cordyceps as a pro-cognitive agent across diverse models.

Notably, most preclinical studies employed chronic administration paradigms (days to weeks) inducing structural adaptations including neurogenesis and mitochondrial biogenesis [10,11] —mechanisms requiring sustained signaling incompatible with acute supplementation. Only Cheng et al. (2025) examined acute effects during exercise stress, reporting that cordycepin preserved learning and memory in mice subjected to exhaustive running [1]. Our human data extend this acute literature by demonstrating faster baseline processing speed with CM—a distinct phenomenon from chronic neuroprotection that may reflect rapid AMPK-mediated metabolic effects rather than structural remodeling [8].

Human evidence for CM’s cognitive effects remains limited. A 2019 systematic review identified three randomized trials on medicinal mushrooms and cognition; only two reported significant improvements in at least one outcome [28]. One trial in middle-aged adults with subjective cognitive decline (12 weeks of CM mycelial extract) found improvements in cognitive assessments and sleep quality [29]. While no prior human study examined acute reaction time effects in healthy young adults, our finding of elevated processing speed aligns with animal evidence of enhanced attentional function following Cordyceps administration.

Cognitive performance, particularly during rest, is closely tied to cerebral energy availability, oxygen delivery, and hemodynamic stability. Our findings suggest that CM may support cognitive function through the following integrated pathways:

Firstly, CM supplementation resulted in a significantly higher resting oxygen uptake (VO₂) and a more stable resting blood glucose profile(although not statistically significant). These metabolic improvements likely contributed to better cognitive performance by ensuring a steady supply of energy substrates to the brain.

Secondly, Although CM did not significantly alter heart rate or blood pressure during rest and exercise, it increased resting VO₂, suggesting improved oxygen extraction and utilization at the tissue level. This enhanced aerobic capacity without excessive cardiovascular demand may have supported cerebral oxygenation. Stable blood pressure and heart rate responses further indicate that CM did not induce autonomic stress, which could otherwise detract from cognitive focus.

Thirdly, the bioactive component cordycepin, an adenosine analog present in CM, is known to activate AMPK—a key regulator of cellular energy homeostasis. AMPK activation promotes mitochondrial biogenesis and oxidative metabolism [1,30], which may explain the observed increases in resting VO₂. In the brain, enhanced mitochondrial efficiency can reduce oxidative stress and support neuronal resilience.

### Effect of cordyceps on cognitive function through blood glucose

Blood glucose declined significantly from pre- to post-exercise in both conditions, reflecting the metabolic demands of exhaustive exercise. However, no significant main effect of Condition or Condition × Time interaction was observed, indicating CM did not differentially influence glycemic responses during this protocol. Pre-exercise glucose values were comparable between conditions, as were post-exercise values.

While maintaining optimal blood glucose levels is critical for cognitive function [31,32], and both hypoglycemia and hyperglycemia can impair attention and reaction time [31], our data provide no evidence that CM modulated glycemia in this acute protocol. This null finding contrasts with chronic Cordyceps administration studies reporting anti-diabetic potential and improved insulin sensitivity [33], highlighting the mechanistic distinction between acute and chronic paradigms. Laboratory studies indicate that Cordyceps extracts can reduce blood sugar levels and improve insulin action through polysaccharides and cordycepin enhancing glucose uptake [33]. However, such metabolic adaptations likely require sustained exposure beyond the 30-minute window employed here. Consequently, while glucose homeostasis theoretically supports cognitive performance [31,32], our data do not support a role for acute CM-induced glycemic modulation in the observed reaction time advantage.

### Effect of cordyceps on cognitive function through blood pressure

Systolic blood pressure increased significantly from pre- to post-exercise in both conditions, reflecting normal hemodynamic responses to maximal exertion. However, no significant main effect of Condition or Condition × Time interaction was observed for systolic pressure. Similarly, diastolic blood pressure showed no significant main effect of condition, main effect of time, or interaction. Pre- and post-exercise values were comparable between conditions for both systolic and diastolic pressure.

Cardiovascular health is intimately linked to brain health, with elevated blood pressure representing a risk factor for cognitive decline through effects on cerebral vasculature [34]. Chronic hypertension contributes to small vessel disease and reduced cerebral perfusion that can impair executive function over time [34]. Even in middle-aged adults, higher blood pressure correlates with lower cognitive performance on tasks of memory and attention in many studies [35], and even in middle-aged adults, higher blood pressure correlates with lower cognitive performance [35]. Acute blood pressure reactivity to stress may also transiently influence cognitive efficiency [34], and aggressive blood pressure control has been associated with slower cognitive decline in at-risk populations [35,36].

Cordyceps has documented antihypertensive and vasodilatory properties in animal models and traditional medicine applications [7], potentially enhancing endothelial function and cerebral perfusion. However, our acute protocol revealed no significant CM-induced alterations in blood pressure responses. This cardiovascular neutrality represents a safety advantage—CM supplementation did not induce hemodynamic stress that could potentially detract from cognitive focus—but provides no evidence that blood pressure modulation contributed to the observed reaction time advantage. The maintenance of stable hemodynamic responses with CM may support cognitive performance indirectly by avoiding excessive autonomic activation, though our data do not permit causal inference regarding this relationship.

### Effect of cordyceps on cognitive function through oxygen uptake

CM supplementation significantly elevated resting oxygen uptake without altering peak exercise VO₂. VO₂ increased substantially from rest to peak exercise in both conditions, with no significant Condition × Time interaction, indicating CM did not differentially modulate the exercise-induced VO₂ trajectory.

The link between physical fitness and cognitive performance is well-established, with higher cardiorespiratory fitness (VO₂max) associated with better performance across multiple cognitive domains including processing speed, working memory, and executive function [37]. Aerobic exercise interventions that raise VO₂max often improve executive functions and attention [38] potentially through enhanced cerebral blood flow, increased neurotrophic factors (e.g., BDNF), and reduced inflammation. Mechanistically, greater aerobic capacity may improve cerebral blood flow, enhance neurotrophic factors (like BDNF), and reduce inflammation – all of which support cognitive performance [37]. Athletes with superior endurance tend to maintain cognitive faculties under fatigue better than less fit individuals, and even modest physical activity improvements correlate with faster reaction times [39].

Several human trials report endurance benefits from chronic Cordyceps supplementation: 12 weeks of Cordyceps sinensis improved 5-km run time and VO₂max in amateur runners [40], and other studies note Cordyceps can lower exercise heart rate and blood lactate with prolonged use [41,42]. These chronic adaptations reflect structural changes including mitochondrial biogenesis [30] requiring sustained AMPK activation over weeks—mechanisms fundamentally distinct from acute effects.

The elevated resting VO₂ observed with acute CM supplementation likely reflects transient metabolic priming—enhanced mitochondrial oxidative efficiency at the tissue level—rather than increased systemic oxygen delivery. Cordycepin activates AMPK within minutes of exposure [43], promoting mitochondrial substrate oxidation and proton leak without requiring structural adaptations such as erythropoiesis [1,30]. While we did not measure hematological parameters (RBC indices, hemoglobin, hematocrit) that would clarify contributions from altered oxygen transport capacity, the acute 30-minute protocol and absence of peak VO₂ enhancement argue against hematological mechanisms. Future studies incorporating complete blood counts, serum iron markers, and direct measures of cerebral/tissue oxygenation (e.g., near-infrared spectroscopy) would help delineate whether acute CM influences oxygen delivery, extraction, or both.

While greater aerobic capacity theoretically supports cerebral oxygenation and cognitive resilience [37], our data show CM elevated resting—but not peak—VO₂, and the absence of a Condition × Time interaction for VO₂ precludes claims that this metabolic effect causally influenced the reaction time advantage. Future studies employing formal mediation analysis are required to test whether acute CM-induced metabolic changes influence cognitive outcomes.

The principal novelty of this study lies in its focus on the acute cognitive-metabolic effects of CM supplementation in humans, a paradigm distinct from the chronic administration models that dominate the preclinical and clinical literature. This is the first randomized controlled trial to demonstrate that a single dose of CM can elevate resting oxygen uptake and improve post-exercise reaction time in healthy young adults, thereby identifying a rapid, potentially AMPK-mediated mechanism that operates independently of long-term structural adaptations. However, several limitations must be acknowledged. The use of a small, homogenous sample (N = 12, recreationally active young men) limits the generalizability of the findings and may have underpowered the detection of smaller interaction effects. The abbreviated 25-trial Stroop task, while pragmatic for the acute post-exercise window, may lack the sensitivity to detect subtler executive function changes. Furthermore, the single post-exercise timepoint (3 min) captures only an immediate snapshot, leaving the temporal dynamics of CM’s effects uncharacterized. Finally, while resting VO₂ was elevated, direct mechanistic evidence—such as measures of cerebral blood flow, neural activation, or plasma cordycepin kinetics—is absent, precluding definitive causal conclusions about the pathway linking CM supplementation to faster cognitive processing. A further limitation is the absence of hematological and biochemical markers of oxygen transport capacity (e.g., hemoglobin, hematocrit, RBC indices, serum ferritin). While the acute protocol and mechanistic rationale favor a metabolic priming interpretation, future trials should include these measures to comprehensively characterize CM’s effects on the oxygen cascade—from pulmonary uptake to mitochondrial utilization.

### Practical implications

The findings of this study offer several actionable insights for athletes, competitive gamers, coaches, and sports nutrition practitioners seeking to optimize integrated physical and cognitive performance.

- **For athletes in time-critical sports:** The 70 ms advantage in reaction time (d = 0.83) observed with acute CM supplementation falls within a range previously associated with meaningful performance differences in competitive sports. In collegiate baseball, reaction time training producing improvements of approximately 133 ms distinguished trained from untrained athletes [44]. In volleyball, training-induced reductions in reaction time of 72–91 ms were accompanied by significant improvements in sport-specific performance metrics, with effect sizes ranging from d = 6.7 to 12.7 [45]. These benchmarks suggest that the 70 ms advantage observed in the present study, while preliminary, is comparable in magnitude to effects previously linked to enhanced athletic performance. Sports requiring rapid decision-making under physical duress (e.g., soccer goalkeeping, tennis returns, basketball defense, baseball batting) may therefore represent contexts where such an advantage could prove meaningful, though direct transfer remains to be demonstrated.
- **For e-sports and competitive gaming:** Sustained attention and rapid response are critical during extended gaming sessions. Acute CM supplementation could theoretically help maintain elevated processing speed without cardiovascular strain, potentially reducing mental fatigue during tournaments. However, no direct evidence yet supports this application.
- **For sports nutrition practitioners:** CM represents a natural supplement option that elevated cognitive processing speed without adverse effects on blood pressure, heart rate, or glucose regulation in this acute protocol. A single 1 g dose administered approximately 30 minutes pre-activity may support contexts requiring sustained rapid decision-making. However, expectations should be tempered: the effect represents baseline performance elevation (a main effect of Condition) rather than protection against exercise-induced impairment, and replication in larger samples is needed before clinical or practical recommendations can be made.
- **For future research:** These results encourage investigation into acute nootropic effects of medicinal fungi, particularly studies employing (a) larger sample sizes to obtain stable effect estimates, (b) multiple post-exercise assessment timepoints to characterize the temporal profile of CM’s cognitive effects, (c) direct measurement of sport-specific transfer (e.g., batting performance, goalkeeper reaction tests), and (d) plasma cordycepin pharmacokinetics to clarify dose-response relationships. Also, subsequent investigations should integrate: (1) pre/post-supplementation complete blood counts with RBC morphology; (2) serum markers of iron metabolism and erythropoietic activity; (3) direct measures of tissue oxygenation (e.g., cerebral NIRS, muscle oximetry); and (4) plasma cordycepin pharmacokinetics to link exposure to physiological response.

## Conclusion

Acute CM supplementation produced a significant improvement in reaction time and elevated resting oxygen uptake without altering blood glucose, blood pressure, or heart rate responses. Exhaustive exercise improved reaction time in both conditions, and CM’s effect manifested as elevated baseline processing speed that persisted through post-exercise assessment. The absence of significant Condition × Time interactions indicates that CM’s effect represents baseline cognitive elevation rather than modulation of exercise-induced changes. These findings suggest acute metabolic priming rather than exercise-specific cognitive modulation, representing a distinct phenomenon from chronic neuroprotective adaptations documented in longer supplementation paradigms. The observed main effect on processing speed provides initial human evidence of acute CM’s influence on cognitive-metabolic parameters—a finding requiring replication and mechanistic validation in future work.

## Supporting information

S1 File Supplementary File S1 CM COA. (DOCX)

S1 Data Farzan. (XLS)
