# Supplementary material for: Acute Cordyceps militaris supplementation and elevated resting oxygen uptake with faster reaction times: A randomized crossover trial
Source: PLoS One. 2026 Jul 15;21(7):e0351725. doi: 10.1371/journal.pone.0351725 (PMC13372127; doi:10.1371/journal.pone.0351725)
Supplement: S1 File — (DOCX) [file pone.0351725.s001.docx]

# Supplementary File S1. Analytical Report for Cordyceps militaris Extract

## Product and Batch Information

• Product name: Cordyceps militaris Fruiting Body Extract

• Manufacturer: Real Mushrooms®

• Product Code: RM-CM150C

• Lot Number: C24156001-1

• Form: Powdered extract in capsule form

• Extract Ratio: 1:1 fruiting body extract

## Third-Party Laboratory Verification (HPLC)

Analytical testing was performed by an independent ISO-certified laboratory using High-Performance Liquid Chromatography (HPLC). The following parameters were verified:

• β-glucan content: ≥150 mg/g (15% w/w)

• Cordycepin content: ≥2.5 mg/g (0.25% w/w)

• Adenosine: Not detected above analytical threshold

• Heavy metals: Below permissible limits (Lead, Arsenic, Cadmium, Mercury)

• Microbiological screening: No pathogenic bacteria detected

## Methods Summary

β-glucans were quantified using an enzymatic HPLC-linked assay designed for polysaccharide characterization.

Cordycepin and other nucleoside analogs were measured using reversed-phase HPLC with UV detection at 260 nm.

All analyses were performed in duplicate, with results meeting product specification criteria.

## Quality and Safety Evaluation

• Moisture content: Within acceptable range for powdered mushroom extracts.

• Appearance: Orange-colored fine powder, consistent with Cordyceps militaris fruiting body extract.

• Odor: Mild, characteristic of mushroom extract.

• Capsule uniformity: Verified visually and by weight consistency testing.

## Notes

This supplementary file provides a study-specific summary of the analytical confirmation of the extract used. The full Certificate of Analysis (COA) is available from the manufacturer (Real Mushrooms®) and was reviewed by the investigators prior to study initiation.
