# Supplementary material for: Acute Cordyceps militaris supplementation and elevated resting oxygen uptake with faster reaction times: A randomized crossover trial
Source: PLoS One. 2026 Jul 15;21(7):e0351725. doi: 10.1371/journal.pone.0351725 (PMC13372127; doi:10.1371/journal.pone.0351725)
Supplement: Attachment — Submitted filename: review 2.doc [file pone.0351725.s003.doc]

PONE-D-26-06035

Acute Cordyceps militaris supplementation and elevated resting oxygen uptake with faster reaction times: a randomized crossover trial

PLOS One

Dear Acadmic editor Dr. Shwet Kamal
 We really appreciate giving us the opportunity of revising manuscript. We also appreciate respected reviewers for all their important comments which ere really helpful to increase the quality of the manuscript. The following items were included when submitting the manuscript:

- A letter that responds to each point raised by the academic editor and reviewer(s). A marked-up copy of your manuscript that highlights changes made to the original version. You should upload this as a separate file labeled 'Revised Manuscript with Track Changes'.
- An unmarked version of revised paper without tracked changes. You should upload this as a separate file labeled 'Manuscript'.

Additional Editor Comments:

The manuscript "Acute Cordyceps militaris supplementation and elevated resting oxygen uptake with faster reaction times: a randomized crossover trial" is an interesting study on effect of Cordyceps militaris on oxygen uptake and cognitive abilities in human respondents. Very few studies on human beings are conducted on the effect of Cordyceps militaris, although there are so many effects are mentioned in the literature.

Surely, the sample size is not enough to draw an absolute conclusion but definitely the study gives an positive indication of the effect of Cordyceps on human body. This study can make a base for the future studies with larger population size. Thus with the minor corrections suggested by the worthy reviewers may be incorporated before it is accepted for publication.

We really appreciate the positive feedback of respected editor and tried our best to consider all required revisions.


Reviewer's Responses to Questions

**Comments to the Author**

1. Is the manuscript technically sound, and do the data support the conclusions?

The manuscript must describe a technically sound piece of scientific research with data that supports the conclusions. Experiments must have been conducted rigorously, with appropriate controls, replication, and sample sizes. The conclusions must be drawn appropriately based on the data presented.

Reviewer #1: Yes

Reviewer #2: Yes

2. Has the statistical analysis been performed appropriately and rigorously?

Reviewer #1: Yes

Reviewer #2: Yes

3. Have the authors made all data underlying the findings in their manuscript fully available?

The [PLOS Data policy](http://track.editorialmanager.com/CL0/http:%2F%2Fwww.plosone.org%2Fstatic%2Fpolicies.action%23sharing/1/010f019d57869994-f21fa842-3a40-4827-baae-f429324cb4dc-000000/TMZ5KlmEOTW6C_P22W5fQ7K2xwSwcLvxGelljtPwc-I=257) requires authors to make all data underlying the findings described in their manuscript fully available without restriction, with rare exception (please refer to the Data Availability Statement in the manuscript PDF file). The data should be provided as part of the manuscript or its supporting information, or deposited to a public repository. For example, in addition to summary statistics, the data points behind means, medians and variance measures should be available. If there are restrictions on publicly sharing data—e.g. participant privacy or use of data from a third party—those must be specified.

Reviewer #1: Yes

Reviewer #2: Yes

4. Is the manuscript presented in an intelligible fashion and written in standard English?

PLOS ONE does not copyedit accepted manuscripts, so the language in submitted articles must be clear, correct, and unambiguous. Any typographical or grammatical errors should be corrected at revision, so please note any specific errors here.

Reviewer #1: Yes

Reviewer #2: Yes

5. Review Comments to the Author

Please use the space provided to explain your answers to the questions above. You may also include additional comments for the author, including concerns about dual publication, research ethics, or publication ethics. (Please upload your review as an attachment if it exceeds 20,000 characters)

Reviewer #1: This manuscript addresses an interesting topic, as human studies on Cordyceps militaris supplementation remain limited. The study provides preliminary evidence that may inform future research with larger cohorts. However, several revisions are necessary to improve clarity, internal consistency, and the strength of the manuscript.

We really appreciate positive feedback and important points raised by respected reviewer and tried our best to consider all points in the revisions of the manuscript.

Major Comments

1.The Results section contains extensive numerical data within the text, which reduces readability. Key statistical outcomes should be summarized in tables to avoid redundancy and improve clarity. This is particularly relevant for the outcomes related to Stroop Test reaction time and Oxygen Uptake at rest, where statistically significant differences from the placebo condition are reported.

We thank the reviewer for this constructive feedback. We agree that the original Results section was overly dense with numerical data, which impeded readability. To address this, we have substantially revised the Results section to improve clarity and reduce redundancy.

Specifically, we have: Created two new summary tables (Table 1 and Table 2) to present the primary cognitive and physiological outcomes. These tables include pre/post means, standard deviations, main effects, interaction effects, and pairwise comparison results (mean differences, p-values, and effect sizes with 95% CIs).

Streamlined the text to focus on the interpretation of the statistical findings (e.g., "Reaction time was significantly faster in the CM condition post-exercise (p = 0.042, d = 0.88)") rather than repeating all F-values and degrees of freedom that are now clearly presented in the tables.

Retained only the most critical statistical details in the text (e.g., the significant main effects for Condition and Time for Stroop RT, and the significant difference for resting VO₂) to guide the reader through the narrative, while directing them to the tables for comprehensive data.

2.Interpretation of Practical Implications
The manuscript suggests that the observed ~70 ms improvement in reaction time may translate into meaningful advantages in sports performance. Given the small sample size, this claim appears somewhat speculative. Additional references supporting the relevance of such reaction time differences for sports performance should be provided.

We thank the reviewer for this important methodological observation. We agree that claims of practical relevance should be grounded in empirical evidence rather than speculation, particularly given our modest sample size. We have revised the "Practical Implications" section accordingly, making three substantive changes.

Minor Comments

Figure 2: The figure legend appears to be missing the symbol (*) indicating statistical significance.

Thank you. Figure 2 was checked and revised.

Figure 4: Numerical values reported in the Results text do not appear fully consistent with those shown in the figure. Please verify and correct if necessary.

Thank you so much for important point. All numbers were checked and modified in text or figures.

Figure 6: Please clarify whether pre-exercise and post-exercise values differ significantly; the large error bars make interpretation unclear.

Thank you . Figure 6 and its caption was modified .

Figure 7: The terminology should be standardized. The term “Recovery” is used here, whereas “Post exercise” is used in Figures 1–6.

We thank the reviewer for this careful observation regarding terminology consistency. We agree that standardized language across all figures is essential for clarity and professionalism. We have corrected this inconsistency as follows: Figure 7 has been revised to replace "Recovery" with "Post-exercise" to match the terminology used in Figures 2, 3, 4, 5, and 6.

Discussion (Page 20): Reduce repetition of statistical values already reported in the Results section and focus more on interpretation.

Thank you for this valuable feedback. We agree that the original Discussion contained excessive repetition of statistical outcomes already detailed in the Results section. The revised version below removes redundant F-values, p-values, exact means/SDs, and confidence intervals, and shifts the focus toward mechanistic interpretation, contextualization with existing literature, and physiological/cognitive implications.

Discussion (Page 21, paragraph 2): Revise the sentence derived from Reference 21 to improve grammatical accuracy.

We thank the reviewer for this careful grammatical observation. The original sentence referencing Yuan et al. (2018) contained a grammatical error in the phrase "Cordyceps militaris polypeptide Condition significantly improved learning and memory performance." The word "Condition" was incorrectly included and capitalized.

We have revised this sentence to read: "In scopolamine-induced amnesia models, Cordyceps militaris polypeptide significantly improved learning and memory performance, likely through antioxidant neuroprotection ."

Acknowledgments: Replace the term “patients” with “volunteers.”

Thank you . it was replaced

Reviewer #2:

We really appreciate positive feedback and important points raised by respected reviewer and tried our best to consider all points in the revisions of the manuscript.

RBC size and some other biochemical features would have included to support increased Oxygen carrying capacity.

Thank you for this insightful comment. We agree that measuring red blood cell (RBC) indices and related biochemical markers would strengthen mechanistic interpretation of the observed elevation in resting oxygen uptake (VO₂). We clarified our interpretive framework, acknowledge this limitation, and propose specific manuscript revisions.

Selected 1g dose its reason of selection and other doses not mentioned.

Thank you for important point. The following information was added to the method section:

The 1 g dose was selected based on: (1) standardization to deliver ≥150 mg β-glucans and ≥2.5 mg cordycepin per serving; (2) alignment with human supplementation literature reporting biological activity of CM extracts within the 1–4 g/day range for acute protocols[20-22]; (3) preclinical pharmacokinetic studies in rats indicating cordycepin undergoes rapid distribution and elimination, with plasma levels declining substantially within ~30 minutes of administration [23]; and (4) practical considerations for tolerability and feasibility within a randomized crossover design..

Participants number is not enough for statistical significance. It is a brief study can be elaborated a lot.

We thank the reviewer for this honest and constructive critique. We agree that the sample size (N=12) is modest and represents a limitation of the present study. We have substantially revised the manuscript to address this concern in three ways.

First, we have provided a more detailed and transparent power analysis in the Methods section. We now explicitly state that while the study was powered to detect large main effects (Cohen's f = 0.35), it was underpowered to detect smaller interaction effects (Condition × Time). We also acknowledge the "winner's curse" phenomenon — the risk that effect sizes from small studies may be inflated.

Second, we have expanded the Limitations section to include a comprehensive discussion of sample size limitations. Third, we have tempered our claims throughout the manuscript — particularly in the Abstract, Conclusion, and Practical Implications — to reflect the preliminary nature of the findings. We now consistently describe the results as "preliminary," "hypothesis-generating," and "requiring replication. This analysis indicates the study was adequately powered to detect main effects of Condition for outcomes with medium-to-large effect sizes—which aligns with our primary findings:

Stroop reaction time: d = 0.83 (95% CI [0.01, 1.65], p = 0.042)

Resting VO₂: d = 0.92 (95% CI [0.24, 1.60], p = 0.022)
