# Supplementary material for: Acute Cordyceps militaris supplementation and elevated resting oxygen uptake with faster reaction times: A randomized crossover trial
Source: PLoS One. 2026 Jul 15;21(7):e0351725. doi: 10.1371/journal.pone.0351725 (PMC13372127; doi:10.1371/journal.pone.0351725)
Supplement: Peer review history

# Peer review: Decision Letter 0

**DOI:** 10.1371/journal.pone.0351725.r001

**Authors:** Shwet Kamal

4 Apr 2026

PONE-D-26-06035

Acute Cordyceps militaris supplementation and elevated resting oxygen uptake with faster reaction times: a randomized crossover trial

PLOS One

Dear Dr. Koushkie Jahromi,

Thank you for submitting your manuscript to PLOS ONE. After careful consideration, we feel that it has merit but does not fully meet PLOS ONE’s publication criteria as it currently stands. Therefore, we invite you to submit a revised version of the manuscript that addresses the points raised during the review process.

Please submit your revised manuscript by May 19 2026 11:59PM. If you will need more time than this to complete your revisions, please reply to this message or contact the journal office at plosone@plos.org. When you're ready to submit your revision, log on to https://www.editorialmanager.com/pone/ and select the 'Submissions Needing Revision' folder to locate your manuscript file.

Please include the following items when submitting your revised manuscript:

- A letter that responds to each point raised by the academic editor and reviewer(s). You should upload this letter as a separate file labeled 'Response to Reviewers'.
- A marked-up copy of your manuscript that highlights changes made to the original version. You should upload this as a separate file labeled 'Revised Manuscript with Track Changes'.
- An unmarked version of your revised paper without tracked changes. You should upload this as a separate file labeled 'Manuscript'.

If you would like to make changes to your financial disclosure, please include your updated statement in your cover letter. Guidelines for resubmitting your figure files are available below the reviewer comments at the end of this letter.

If applicable, we recommend that you deposit your laboratory protocols in protocols.io to enhance the reproducibility of your results. Protocols.io assigns your protocol its own identifier (DOI) so that it can be cited independently in the future. For instructions see: https://journals.plos.org/plosone/s/submission-guidelines#loc-laboratory-protocols. Additionally, PLOS ONE offers an option for publishing peer-reviewed Lab Protocol articles, which describe protocols hosted on protocols.io. Read more information on sharing protocols at https://plos.org/protocols?utm_medium=editorial-email&utm_source=authorletters&utm_campaign=protocols.

As the corresponding author, your ORCID iD is verified in the submission system and will appear in the published article. PLOS supports the use of ORCID, and we encourage all coauthors to register for an ORCID iD and use it as well. Please encourage your coauthors to verify their ORCID iD within the submission system before final acceptance, as unverified ORCID iDs will not appear in the published article. *Only*   the individual author can complete the verification step; PLOS staff *cannot*   verify ORCID iDs on behalf of authors.

We look forward to receiving your revised manuscript.

Kind regards,

Shwet Kamal, Ph.D

Academic Editor

PLOS One

Journal Requirements:

When submitting your revision, we need you to address these additional requirements.

1.Please ensure that your manuscript meets PLOS ONE's style requirements, including those for file naming. The PLOS ONE style templates can be found at

https://journals.plos.org/plosone/s/file?id=wjVg/PLOSOne_formatting_sample_main_body.pdf and

https://journals.plos.org/plosone/s/file?id=ba62/PLOSOne_formatting_sample_title_authors_affiliations.pdf

2. Please include captions for your Supporting Information files at the end of your manuscript, and update any in-text citations to match accordingly. Please see our Supporting Information guidelines for more information: http://journals.plos.org/plosone/s/supporting-information.

3. If the reviewer comments include a recommendation to cite specific previously published works, please review and evaluate these publications to determine whether they are relevant and should be cited. There is no requirement to cite these works unless the editor has indicated otherwise.

4. Please review your reference list to ensure that it is complete and correct. If you have cited papers that have been retracted, please include the rationale for doing so in the manuscript text, or remove these references and replace them with relevant current references. Any changes to the reference list should be mentioned in the rebuttal letter that accompanies your revised manuscript. If you need to cite a retracted article, indicate the article’s retracted status in the References list and also include a citation and full reference for the retraction notice.

Additional Editor Comments:

The manuscript "Acute Cordyceps militaris supplementation and elevated resting oxygen uptake with faster reaction times: a randomized crossover trial" is an interesting study on effect of Cordyceps militaris on oxygen uptake and cognitive abilities in human respondents. Very few studies on human beings are conducted on the effect of Cordyceps militaris, although there are so many effects are mentioned in the literature.

Surely, the sample size is not enough to draw an absolute conclusion but definitely the study gives an positive indication of the effect of Cordyceps on human body. This study can make a base for the future studies with larger population size. Thus with the minor corrections suggested by the worthy reviewers may be incorporated before it is accepted for publication.

[Note: HTML markup is below. Please do not edit.]

Reviewer's Responses to Questions

**Comments to the Author**

1. Is the manuscript technically sound, and do the data support the conclusions?

The manuscript must describe a technically sound piece of scientific research with data that supports the conclusions. Experiments must have been conducted rigorously, with appropriate controls, replication, and sample sizes. The conclusions must be drawn appropriately based on the data presented.

Reviewer #1: Yes

Reviewer #2: Yes

**********

2. Has the statistical analysis been performed appropriately and rigorously?

Reviewer #1: Yes

Reviewer #2: Yes

**********

3. Have the authors made all data underlying the findings in their manuscript fully available?

The PLOS Data policy requires authors to make all data underlying the findings described in their manuscript fully available without restriction, with rare exception (please refer to the Data Availability Statement in the manuscript PDF file). The data should be provided as part of the manuscript or its supporting information, or deposited to a public repository. For example, in addition to summary statistics, the data points behind means, medians and variance measures should be available. If there are restrictions on publicly sharing data—e.g. participant privacy or use of data from a third party—those must be specified.

Reviewer #1: Yes

Reviewer #2: Yes

**********

4. Is the manuscript presented in an intelligible fashion and written in standard English?

PLOS ONE does not copyedit accepted manuscripts, so the language in submitted articles must be clear, correct, and unambiguous. Any typographical or grammatical errors should be corrected at revision, so please note any specific errors here.

Reviewer #1: Yes

Reviewer #2: Yes

**********

5. Review Comments to the Author

Please use the space provided to explain your answers to the questions above. You may also include additional comments for the author, including concerns about dual publication, research ethics, or publication ethics. (Please upload your review as an attachment if it exceeds 20,000 characters)

Reviewer #1: This manuscript addresses an interesting topic, as human studies on Cordyceps militaris supplementation remain limited. The study provides preliminary evidence that may inform future research with larger cohorts. However, several revisions are necessary to improve clarity, internal consistency, and the strength of the manuscript.

Major Comments

1.The Results section contains extensive numerical data within the text, which reduces readability. Key statistical outcomes should be summarized in tables to avoid redundancy and improve clarity. This is particularly relevant for the outcomes related to Stroop Test reaction time and Oxygen Uptake at rest, where statistically significant differences from the placebo condition are reported.

2.Interpretation of Practical Implications

The manuscript suggests that the observed ~70 ms improvement in reaction time may translate into meaningful advantages in sports performance. Given the small sample size, this claim appears somewhat speculative. Additional references supporting the relevance of such reaction time differences for sports performance should be provided.

Minor Comments

Figure 2: The figure legend appears to be missing the symbol (*) indicating statistical significance.

Figure 4: Numerical values reported in the Results text do not appear fully consistent with those shown in the figure. Please verify and correct if necessary.

Figure 6: Please clarify whether pre-exercise and post-exercise values differ significantly; the large error bars make interpretation unclear.

Figure 7: The terminology should be standardized. The term “Recovery” is used here, whereas “Post exercise” is used in Figures 1–6.

Discussion (Page 20): Reduce repetition of statistical values already reported in the Results section and focus more on interpretation.

Discussion (Page 21, paragraph 2): Revise the sentence derived from Reference 21 to improve grammatical accuracy.

Acknowledgments: Replace the term “patients” with “volunteers.”

Reviewer #2: RBC size and some other biochemical features would have included to support increased Oxygen carrying capacity. Selected 1g dose its reason of selection and other doses not mentioned. Participants number is not enough for statistical significance. It is a brief study can be elaborated a lot.

**********

6. PLOS authors have the option to publish the peer review history of their article (what does this mean?). If published, this will include your full peer review and any attached files.

If you choose “no”, your identity will remain anonymous but your review may still be made public.

**Do you want your identity to be public for this peer review?**   For information about this choice, including consent withdrawal, please see our Privacy Policy.

Reviewer #1: No

Reviewer #2: **Yes:**   Dr Chand Pasha

**********

[NOTE: If reviewer comments were submitted as an attachment file, they will be attached to this email and accessible via the submission site. Please log into your account, locate the manuscript record, and check for the action link "View Attachments". If this link does not appear, there are no attachment files.]

To ensure your figures meet our technical requirements, please review our figure guidelines: https://journals.plos.org/plosone/s/figures

You may also use PLOS’s free figure tool, NAAS, to help you prepare publication quality figures: https://journals.plos.org/plosone/s/figures#loc-tools-for-figure-preparation.

NAAS will assess whether your figures meet our technical requirements by comparing each figure against our figure specifications.

# Author comment: Author response to Decision Letter 1

**DOI:** 10.1371/journal.pone.0351725.r002

14 Apr 2026

PONE-D-26-06035

Acute Cordyceps militaris supplementation and elevated resting oxygen uptake with faster reaction times: a randomized crossover trial

PLOS One

Dear Acadmic editor Dr. Shwet Kamal

We really appreciate giving us the opportunity of revising manuscript. We also appreciate respected reviewers for all their important comments which ere really helpful to increase the quality of the manuscript. The following items were included when submitting the manuscript:

• A letter that responds to each point raised by the academic editor and reviewer(s). A marked-up copy of your manuscript that highlights changes made to the original version. You should upload this as a separate file labeled 'Revised Manuscript with Track Changes'.

• An unmarked version of revised paper without tracked changes. You should upload this as a separate file labeled 'Manuscript'.

Additional Editor Comments:

The manuscript "Acute Cordyceps militaris supplementation and elevated resting oxygen uptake with faster reaction times: a randomized crossover trial" is an interesting study on effect of Cordyceps militaris on oxygen uptake and cognitive abilities in human respondents. Very few studies on human beings are conducted on the effect of Cordyceps militaris, although there are so many effects are mentioned in the literature.

Surely, the sample size is not enough to draw an absolute conclusion but definitely the study gives an positive indication of the effect of Cordyceps on human body. This study can make a base for the future studies with larger population size. Thus with the minor corrections suggested by the worthy reviewers may be incorporated before it is accepted for publication.

We really appreciate the positive feedback of respected editor and tried our best to consider all required revisions.

5. Review Comments to the Author

Please use the space provided to explain your answers to the questions above. You may also include additional comments for the author, including concerns about dual publication, research ethics, or publication ethics. (Please upload your review as an attachment if it exceeds 20,000 characters)

Reviewer #1: This manuscript addresses an interesting topic, as human studies on Cordyceps militaris supplementation remain limited. The study provides preliminary evidence that may inform future research with larger cohorts. However, several revisions are necessary to improve clarity, internal consistency, and the strength of the manuscript.

We really appreciate positive feedback and important points raised by respected reviewer and tried our best to consider all points in the revisions of the manuscript.

Major Comments

1.The Results section contains extensive numerical data within the text, which reduces readability. Key statistical outcomes should be summarized in tables to avoid redundancy and improve clarity. This is particularly relevant for the outcomes related to Stroop Test reaction time and Oxygen Uptake at rest, where statistically significant differences from the placebo condition are reported.

We thank the reviewer for this constructive feedback. We agree that the original Results section was overly dense with numerical data, which impeded readability. To address this, we have substantially revised the Results section to improve clarity and reduce redundancy.

Specifically, we have: Created two new summary tables (Table 1 and Table 2) to present the primary cognitive and physiological outcomes. These tables include pre/post means, standard deviations, main effects, interaction effects, and pairwise comparison results (mean differences, p-values, and effect sizes with 95% CIs).

Streamlined the text to focus on the interpretation of the statistical findings (e.g., "Reaction time was significantly faster in the CM condition post-exercise (p = 0.042, d = 0.88)") rather than repeating all F-values and degrees of freedom that are now clearly presented in the tables.

Retained only the most critical statistical details in the text (e.g., the significant main effects for Condition and Time for Stroop RT, and the significant difference for resting VO₂) to guide the reader through the narrative, while directing them to the tables for comprehensive data.

2.Interpretation of Practical Implications

The manuscript suggests that the observed ~70 ms improvement in reaction time may translate into meaningful advantages in sports performance. Given the small sample size, this claim appears somewhat speculative. Additional references supporting the relevance of such reaction time differences for sports performance should be provided.

We thank the reviewer for this important methodological observation. We agree that claims of practical relevance should be grounded in empirical evidence rather than speculation, particularly given our modest sample size. We have revised the "Practical Implications" section accordingly, making three substantive changes.

Minor Comments

Figure 2: The figure legend appears to be missing the symbol (*) indicating statistical significance.

Thank you. Figure 2 was checked and revised.

Figure 4: Numerical values reported in the Results text do not appear fully consistent with those shown in the figure. Please verify and correct if necessary.

Thank you so much for important point. All numbers were checked and modified in text or figures.

Figure 6: Please clarify whether pre-exercise and post-exercise values differ significantly; the large error bars make interpretation unclear.

Thank you . Figure 6 and its caption was modified .

Figure 7: The terminology should be standardized. The term “Recovery” is used here, whereas “Post exercise” is used in Figures 1–6.

We thank the reviewer for this careful observation regarding terminology consistency. We agree that standardized language across all figures is essential for clarity and professionalism. We have corrected this inconsistency as follows: Figure 7 has been revised to replace "Recovery" with "Post-exercise" to match the terminology used in Figures 2, 3, 4, 5, and 6.

Discussion (Page 20): Reduce repetition of statistical values already reported in the Results section and focus more on interpretation.

Thank you for this valuable feedback. We agree that the original Discussion contained excessive repetition of statistical outcomes already detailed in the Results section. The revised version below removes redundant F-values, p-values, exact means/SDs, and confidence intervals, and shifts the focus toward mechanistic interpretation, contextualization with existing literature, and physiological/cognitive implications.

Discussion (Page 21, paragraph 2): Revise the sentence derived from Reference 21 to improve grammatical accuracy.

We thank the reviewer for this careful grammatical observation. The original sentence referencing Yuan et al. (2018) contained a grammatical error in the phrase "Cordyceps militaris polypeptide Condition significantly improved learning and memory performance." The word "Condition" was incorrectly included and capitalized.

We have revised this sentence to read: "In scopolamine-induced amnesia models, Cordyceps militaris polypeptide significantly improved learning and memory performance, likely through antioxidant neuroprotection ."

Acknowledgments: Replace the term “patients” with “volunteers.”

Thank you . it was replaced

Reviewer #2:

We really appreciate positive feedback and important points raised by respected reviewer and tried our best to consider all points in the revisions of the manuscript.

RBC size and some other biochemical features would have included to support increased Oxygen carrying capacity.

Thank you for this insightful comment. We agree that measuring red blood cell (RBC) indices and related biochemical markers would strengthen mechanistic interpretation of the observed elevation in resting oxygen uptake (VO₂). We clarified our interpretive framework, acknowledge this limitation, and propose specific manuscript revisions.

Selected 1g dose its reason of selection and other doses not mentioned.

Thank you for important point. The following information was added to the method section:

The 1 g dose was selected based on: (1) standardization to deliver ≥150 mg β-glucans and ≥2.5 mg cordycepin per serving; (2) alignment with human supplementation literature reporting biological activity of CM extracts within the 1–4 g/day range for acute protocols[20-22]; (3) preclinical pharmacokinetic studies in rats indicating cordycepin undergoes rapid distribution and elimination, with plasma levels declining substantially within ~30 minutes of administration [23]; and (4) practical considerations for tolerability and feasibility within a randomized crossover design..

Participants number is not enough for statistical significance. It is a brief study can be elaborated a lot.

We thank the reviewer for this honest and constructive critique. We agree that the sample size (N=12) is modest and represents a limitation of the present study. We have substantially revised the manuscript to address this concern in three ways.

First, we have provided a more detailed and transparent power analysis in the Methods section. We now explicitly state that while the study was powered to detect large main effects (Cohen's f = 0.35), it was underpowered to detect smaller interaction effects (Condition × Time). We also acknowledge the "winner's curse" phenomenon — the risk that effect sizes from small studies may be inflated.

Second, we have expanded the Limitations section to include a comprehensive discussion of sample size limitations. Third, we have tempered our claims throughout the manuscript — particularly in the Abstract, Conclusion, and Practical Implications — to reflect the preliminary nature of the findings. We now consistently describe the results as "preliminary," "hypothesis-generating," and "requiring replication. This analysis indicates the study was adequately powered to detect main effects of Condition for outcomes with medium-to-large effect sizes—which aligns with our primary findings:

Stroop reaction time: d = 0.83 (95% CI [0.01, 1.65], p = 0.042)

Resting VO₂: d = 0.92 (95% CI [0.24, 1.60], p = 0.022)

Attachment Submitted filename: review 2.doc

# Peer review: Decision Letter 1

**DOI:** 10.1371/journal.pone.0351725.r003

**Authors:** Shwet Kamal

1 Jun 2026

Acute Cordyceps militaris supplementation and elevated resting oxygen uptake with faster reaction times: a randomized crossover trial

PONE-D-26-06035R1

Dear Dr. Koushkie Jahromi,

We’re pleased to inform you that your manuscript has been judged scientifically suitable for publication and will be formally accepted for publication once it meets all outstanding technical requirements.

Within one week, you’ll receive an e-mail detailing the required amendments. When these have been addressed, you’ll receive a formal acceptance letter and your manuscript will be scheduled for publication.

An invoice will be generated when your article is formally accepted. Please note, if your institution has a publishing partnership with PLOS and your article meets the relevant criteria, all or part of your publication costs will be covered. Please make sure your user information is up-to-date by logging into Editorial Manager at Editorial Manager® and clicking the ‘Update My Information' link at the top of the page. For questions related to billing, please contact billing support.

If your institution or institutions have a press office, please notify them about your upcoming paper to help maximize its impact. If they’ll be preparing press materials, please inform our press team as soon as possible -- no later than 48 hours after receiving the formal acceptance. Your manuscript will remain under strict press embargo until 2 pm Eastern Time on the date of publication. For more information, please contact onepress@plos.org.

Kind regards,

Shwet Kamal, Ph.D

Academic Editor

PLOS One

Additional Editor Comments (optional):

The manuscript entitled "Acute Cordyceps militaris supplementation and elevated resting oxygen uptake with faster reaction times: a randomized crossover trial" may be accepted.

Reviewers' comments:

Reviewer's Responses to Questions

**Comments to the Author**

1. If the authors have adequately addressed your comments raised in a previous round of review and you feel that this manuscript is now acceptable for publication, you may indicate that here to bypass the “Comments to the Author” section, enter your conflict of interest statement in the “Confidential to Editor” section, and submit your "Accept" recommendation.

Reviewer #1: All comments have been addressed

Reviewer #2: All comments have been addressed

**********

2. Is the manuscript technically sound, and do the data support the conclusions?

The manuscript must describe a technically sound piece of scientific research with data that supports the conclusions. Experiments must have been conducted rigorously, with appropriate controls, replication, and sample sizes. The conclusions must be drawn appropriately based on the data presented.

Reviewer #1: Yes

Reviewer #2: Yes

**********

3. Has the statistical analysis been performed appropriately and rigorously?

Reviewer #1: Yes

Reviewer #2: Yes

**********

4. Have the authors made all data underlying the findings in their manuscript fully available?

The PLOS Data policy requires authors to make all data underlying the findings described in their manuscript fully available without restriction, with rare exception (please refer to the Data Availability Statement in the manuscript PDF file). The data should be provided as part of the manuscript or its supporting information, or deposited to a public repository. For example, in addition to summary statistics, the data points behind means, medians and variance measures should be available. If there are restrictions on publicly sharing data—e.g. participant privacy or use of data from a third party—those must be specified.

Reviewer #1: (No Response)

Reviewer #2: Yes

**********

5. Is the manuscript presented in an intelligible fashion and written in standard English?

PLOS ONE does not copyedit accepted manuscripts, so the language in submitted articles must be clear, correct, and unambiguous. Any typographical or grammatical errors should be corrected at revision, so please note any specific errors here.

Reviewer #1: Yes

Reviewer #2: Yes

**********

6. Review Comments to the Author

Please use the space provided to explain your answers to the questions above. You may also include additional comments for the author, including concerns about dual publication, research ethics, or publication ethics. (Please upload your review as an attachment if it exceeds 20,000 characters)

Reviewer #1: The authors have adequately addressed the reviewer comments and revised the manuscript accordingly. In its current form, the manuscript is suitable for publication.

Reviewer #2: Authors did not addressed by doing experiments like RBC size and increasing sample size. But mentioned technical inability.

**********

7. PLOS authors have the option to publish the peer review history of their article (what does this mean?). If published, this will include your full peer review and any attached files.

If you choose “no”, your identity will remain anonymous but your review may still be made public.

**Do you want your identity to be public for this peer review?**   For information about this choice, including consent withdrawal, please see our Privacy Policy.

Reviewer #1: No

Reviewer #2: No

**********

# Editor report: Acceptance letter

**DOI:** 10.1371/journal.pone.0351725.r004

**Authors:** Shwet Kamal

PONE-D-26-06035R1

PLOS One

Dear Dr. Koushkie Jahromi,

I'm pleased to inform you that your manuscript has been deemed suitable for publication in PLOS One. Congratulations! Your manuscript is now being handed over to our production team.

At this stage, our production department will prepare your paper for publication. This includes ensuring the following:

* All references, tables, and figures are properly cited

* All relevant supporting information is included in the manuscript submission,

* There are no issues that prevent the paper from being properly typeset

You will receive further instructions from the production team, including instructions on how to review your proof when it is ready. Please keep in mind that we are working through a large volume of accepted articles, so please give us a few days to review your paper and let you know the next and final steps.

Lastly, if your institution or institutions have a press office, please let them know about your upcoming paper now to help maximize its impact. If they'll be preparing press materials, please inform our press team within the next 48 hours. Your manuscript will remain under strict press embargo until 2 pm Eastern Time on the date of publication. For more information, please contact onepress@plos.org.

You will receive an invoice from PLOS for your publication fee after your manuscript has reached the completed accept phase. If you receive an email requesting payment before acceptance or for any other service, this may be a phishing scheme. Learn how to identify phishing emails and protect your accounts at https://explore.plos.org/phishing.

If we can help with anything else, please email us at customercare@plos.org.

Thank you for submitting your work to PLOS ONE and supporting open access.

Kind regards,

PLOS ONE Editorial Office Staff

on behalf of

Dr. Shwet Kamal

Academic Editor

PLOS One
